# Fluorescent Soybean Hairy Root Construction and Its Application in the Soybean—Nematode Interaction: An Investigation

**DOI:** 10.3390/biology10121353

**Published:** 2021-12-20

**Authors:** Ruowei Yang, Shuang Li, Xiaowen Yang, Xiaofeng Zhu, Haiyan Fan, Yuanhu Xuan, Lijie Chen, Xiaoyu Liu, Yuanyuan Wang, Yuxi Duan

**Affiliations:** 1 College of Plant Protection, Shenyang Agricultural University, Shenyang 110866, China; yrw@syau.edu.cn (R.Y.); 2020200130@stu.syau.edu.cn (X.Y.); syxf2000@syau.edu.cn (X.Z.); fanhaiyan6860@163.com (H.F.); xuanyuanhu115@syau.edu.cn (Y.X.); chenlj-0210@syau.edu.cn (L.C.); 2 Shaanxi Key Laboratory of Chinese Jujube, Yan’an University, Yan’an 716000, China; shuangli@yau.edu.cn; 3 College of Science, Shenyang Agricultural University, Shenyang 110866, China; liuxiaoyu7805@163.com; 4 College of Biotechnology, Shenyang Agricultural University, Shenyang 110866, China

**Keywords:** soybean, soybean cyst nematode, resistance gene, interaction, methodology

## Abstract

**Simple Summary:**

The soybean cyst nematode is a pathogen that is parasitic on soybean roots and causes high yield losses. To control it, it is necessary to study resistance genes and their mechanisms. The existing means take half a year but our new method can accelerate the process. We built new tools and integrated the advantages of current technologies to develop an FHR-SCN system. This method shortens the experimental period from half a year to six weeks. Researchers can differentiate between the roots that are transgenic and those that are not with a blue light flashlight and filter. Using this method, we verified a gene that could provide an additional contribution to resistance against the nematode. In addition, we used a transgenic soybean to verify and further indicate that this resistance was caused by an increase of jasmonic acid. The FHR-SCN pathosystem will accelerate the study of the soybean resistant gene.

**Abstract:**

Background: The yield of soybean is limited by the soybean cyst nematode (SCN, *Heterodera glycines*). Soybean transformation plays a key role in gene function research but the stable genetic transformation of soybean usually takes half a year. Methods: Here, we constructed a vector, pNI-GmUbi, in an *Agrobacterium rhizogenes*-mediated soybean hypocotyl transformation to induce fluorescent hairy roots (FHRs). Results: We describe the operation of FHR-SCN, a fast, efficient and visual operation pathosystem to study the gene functions in the soybean-SCN interaction. With this method, FHRs were detected after 25 days in 4 cultivars (Williams 82, Zhonghuang 13, Huipizhiheidou and Peking) and at least 66.67% of the composite plants could be used to inoculate SCNs. The demographics of the SCN could be started 12 days post-SCN inoculation. Further, *GmHS1^pro-1^* was overexpressed in the FHRs and *GmHS1^pro-1^* provided an additional resistance in Williams 82. In addition, we found that jasmonic acid and JA-Ile increased in the transgenic soybean, implying that the resistance was mainly caused by affecting the content of JA and JA-Ile. Conclusions: In this study, we established a pathosystem, FHR-SCN, to verify the functional genes in soybeans and the SCN interaction. We also verified that *GmHS1^pro-1^* provides additional resistance in both FHRs and transgenic soybeans, and the resistance may be caused by an increase in JA and JA-Ile contents.

## 1. Introduction

The soybean (*Glycine max* (L.) Merr.) is one of the most important crop plants worldwide and is a major source of oil and proteins but the yield is restricted by the soybean cyst nematode (SCN, *Heterodera glycines*), which is parasitic on the soybean root. The main means to rescue the yield losses caused by SCNs is genetic resistance [1]. The resistance of most commercial resistant cultivars comes from Peking and PI88788 but are being overcome gradually by the SCN [2].

In recent years, the functional analysis of *rhg1* and *Rhg4* has achieved great success [3]. Four resistance genes within them have been gradually verified including *GmAAT* [4], *GmSNAP18* [5], *GmWI12* [6] and *GmSHMT08* [7]. Two types of resistance phenotype were found within those genes, the Peking type and the PI88788 type [2]. The Peking type causes a rapid resistance response to SCN race 3 [8,9] whereas the PI88788 type causes syncytium degeneration slowly [10]. Both resistance types have led to the developmental arrest of SCN juveniles, which have been observed with acid fuchsin staining [11]. The resistance gene commonly inhibits the development of SCNs but the infection process is not restrained. These results have encouraged the studies of resistance candidate genes, such as leucine-rich region (LRR)-containing proteins, protein kinases [12], cytochrome P450s [13] and enzymes in the secondary metabolic pathway [14,15].

Soybean transformation has played a critical role in gene function research [16,17] but the stable genetic transformation of soybean usually takes four to six months to harvest transgenic plants. Therefore, it is necessary to develop an efficient gene expression system to study the resistant genes.

Compared with genetic transformation, *Agrobacterium rhizogenes* (*A. rhizogenes*)-mediated hairy root transformation is more rapid and has a higher transformation frequency [18]. *A. rhizogenes* causes the hairy root of soybean in a manner similar to the crown gall caused by *Agrobacterium tumefaciens*. Both infect at wound sites and transfer T-DNA from the bacteria to the soybean cell. Initially, hairy roots were induced from cotyledon explants with *A. rhizogenes* strain K599. The Integrative Legume Research Group (IGRS, Australia) provided a modified protocol, which allowed hairy root to be regenerated from the cotyledonary node and hypocotyl [18]. Due to SCN parasites in soybean roots, it is convenient to verify the resistance of target genes against SCNs in hairy roots [19,20].

These cases and methods have accelerated the research of soybean root biology but there are three shortcomings with the use of an *A. rhizogenes*-mediated hairy root transformation to study soybean and SCN interactions. The first is the cumbersome process of identifying positive adventitious roots via PCR or Sanger sequencing, which are direct but inefficient. One of the solutions is to use anthocyanins as markers, which have been applied to the study of root nodules [21]. However, anthocyanin belongs to flavonoids that may not suitable for the study of the interaction between soybeans and *H. glycines* due to the resistance potential of anthocyanins [21,22,23]. GFP was considered as a fluorescent marker in a soybean transgenic selection [24]. The second shortcoming is that most operations require a sterile environment, which seriously affects the efficiency although this can be solved by using hypocotyl at the *Agrobacterium* infection site. The last shortcoming is that second stage juveniles (J2s, the infectious stage) of *H. glycines* need to be sterilized before inoculation. This operation reduces the vitality of the nematodes.

Here, we report a rapid, visible and efficient fluorescent hairy root versus an SCN (FHR-SCN) pathosystem for the verification of the resistance phenotype of the candidate genes in soybeans. The FHR-SCN pathosystem is based on the *A. rhizogenes*-mediated soybean hypocotyl transformation but does not dependent on an aseptic operation; there is also no need to prepare a co-culture medium and a root induction medium or to sterilize the SCN juveniles. Positive roots can be selected visually and inoculated with SCNs. With this method, it only took 6 weeks to verify the phenotype of the candidate gene. Further, we constructed a novel plasmid, pNI-GmUbi, for overexpression experiments. In addition, the resistant phenotype of *GmHS1^pro-1^* was reproduced from transgenic soybean plants with the FHR-SCN method. The results showed that there was little difference between the two methods.

In conclusion, the FHR-SCN pathosystem greatly reduces the time to verify resistance phenotypes against SCNs. We anticipate that the pathosystem will be widely applied to the analysis of the interactions between soybeans and SCNs.

## 2. Materials and Methods

### 2.1. Plant and Nematode Materials

Two soybean resistant varieties, Williams 82 (W82) [25] and Zhonghuang 13 (Z13) [26], and two susceptible varieties, Peking [27] and Huipizhiheidou (HPZ, ZDD2315) [28], were used for the *A. rhizogenes* strain K599-mediated fluorescent hairy root transformation. Soybean plants were grown at 27 °C with a 10 h photoperiod in a greenhouse. *H. glycines* race 3, one of the most widely distributed races in China, was tested in this study. The soybean cultivars and SCNs mentioned above were provided by the Nematology Institute of Northern China (Shenyang Agricultural University, Shenyang, China).

*GmHS1^pro-1^* overexpressed Williams 82 soybean transgenic lines were induced via an *A. tumefaciens*-mediated transformation. The T2 generation soybean seeds were provided by the College of Life Science (Yan’an University, Yan’an, China). The transgenic soybean was used to assay the resistant phenotype compared with the control group, wild-type Williams 82.

### 2.2. A. Rhizogenes Strain K599 Materials

The wild-type *A. rhizogenes* strain K599 was provided by the Integrative Legume Research Group (The University of Queensland, Brisbane, Australia). The *A. rhizogenes* strain K599 competent cell preparation and transformation followed the modified freeze–thaw method [29]. A positive transformed single colony was picked from the plate and cultivated in 10 mL LB broth (50 μg/mL kanamycin) at 250 rpm for 24 h at 28 °C. A PCR assay with a specific cloning primer pair was then used to check whether the colony was positive. The positive bacterial culture was stored at 4 °C. One day before the hairy root transformation, a streak of 0.1 mL bacteria was cultivated onto the surface of LB agar plates containing 50 μg/mL kanamycin and cultured for 24 h at 28 °C.

### 2.3. A. Rhizogenes-Mediated Soybean Hypocotyl Transformation

Soybean cultivars Williams 82, Zhonghuang 13, Peking and Huipizhiheidou were used to detect the transformation efficiency. Soybean cultivar Williams 82 was used to induce transgenic FHRs and the empty vector control.

**Figure 1:**
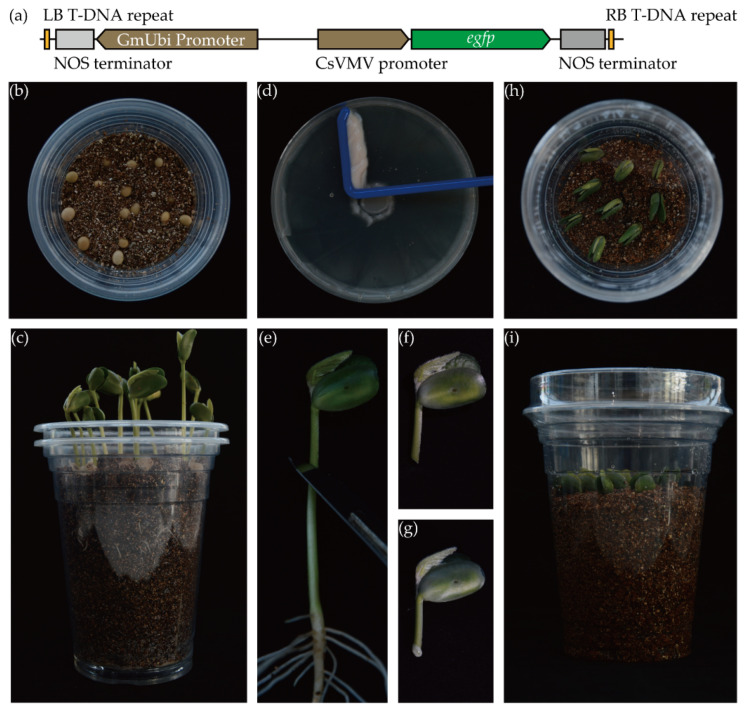
The transformation procedure and culture of soybean hairy roots in vermiculite. (**a**) Diagram of T-DNA region in binary vector pNI-GmUbi; (**b**) density of soybean seeds in a disposable cup: 15 seeds in one 400 mL cup are recommended; (**c**) five-day-old soybean seedlings; (**d**) bacterial mass agglomerated by a spreader in a 9 cm Petri dish; (**e**) the seedling was cut obliquely with a scalpel and aboveground part is used as an explant (**f**); a drop of bacterial mass was immediately applied onto the wound (**g**); (**h**) explants were inserted vertically into vermiculite: 9 explants in 1 400 mL cup are recommended; (**i**) vermiculite was wet and covered the bottom of the Petri dish to maintain humidity.

The soybean seeds were placed in vermiculite and germinated in a greenhouse for 5 days. The primary roots of 5-day-old soybean seedlings were obliquely cut off near the hypocotyl with a sterile blade. A drop of bacterial mass was applied to the wound of the explant (the key steps of the procedure are illustrated in Figure 1e–g.). The explants were then transplanted into wet vermiculite (in disposable drink cups) vertically and the cups were covered to maintain a high humidity.

The fluorescence in the positive hairy roots was excited by a LUYOR-3415RG blue light flashlight (Luyor, Shanghai, China). The roots emitted a green fluorescence that could be observed through a bind pass filter (λ_0_ ≈ 495 nm, Luyor, Shanghai, China). The composite soybean plants were washed after 20 days of growth for the fluorescence detection. The number of regenerated roots and fluorescent hairy roots (FHRs) of 30 composite plants in each soybean cultivar were counted. The results were plotted and the significant differences were analyzed using a one-way ANOVA (GraphPad PRISM software, San Diago, CA, USA).

### 2.4. Incubation and Inoculation of SCNs

Cysts of *H. glycines* SCN race 3 were primarily isolated from SCN infested soil. The soil was added into 2 times the volume of water, fully stirred and precipitated for 30 s to separate the soil and float the cysts. The supernatant was filtered through 420 μm and 180 μm sieves. This process was repeated at least 3 times to collect the cysts on 180 μm sieves completely. The cysts were ground with a rubber tube stopper on the 180 μm sieve and the eggs were re-collected on a 25 μm sieve. The eggs were resuspended with a 35% sucrose solution, stirred and centrifuged for 1 min at 3000 rpm for purification. The eggs were then soaked in a 0.1% NaClO solution to sterilize and hatched with 3 mM ZnSO_4_ at 27 °C avoiding light. The hatched second stage juveniles (J2s) were collected every day and precipitated in beakers at 4 °C avoiding light.

The supernatant was carefully removed to enrich the J2s, which were then counted under an SMZ800 stereomicroscope (Nikon, Japan) until the concentration of J2s was 1000 pcs/mL. This was diluted 10 times with sterilized 0.2% water-agar to 100 pcs/mL. Before inoculation, 5 mL of the J2s suspension was laid at the bottom of 15 mL centrifuge tubes. The negative roots of composite plant were then removed and the whole roots were soaked in the J2s suspension. The plants were grown under a weak light and the roots were kept away from light for 2 days, then transferred to wet vermiculite for another 10 days of growth.

### 2.5. SCN Demographic Assays

After 12 days post-inoculation (dpi), the roots were stained using the acid fuchsin method [11] and the SCNs were counted in different development stages [3].

### 2.6. Construction of the Plasmids

To build the plasmid pNI-GmUbi, three elements including *egfp* ORF, a CsVMV promoter and a GmUbi promoter were synthesized and cloned into two clone vectors by Genewiz (https://www.genewiz.com.cn/, accessed on 23 December 2019). The backbone of pNI-GmUbi was amplified by PCR reactions from pCAMBIA1302. The backbone and cassettes were then assembled with an In-Fusion Snap Assembly Mix (TaKaRa, Beijing, China). The schematic diagram of the construction process and the FASTA format sequence of pNI-GmUbi are provided in Supplementary Materials Figure S1. In the pNI-GmUbi plasmid (empty vector), a Kozak sequence and a *Xho* I restriction site were inserted at 3′ of the GmUbi promoter. The purpose of introducing the *Xho* I site was to insert the target gene for the expression.

The primers to clone the ORF of *GmHS1^pro-1^* (Glyma.02g255400) are shown in Supplementary Materials Table S1. The plasmid pNI-GmUbi was linearized by a QuickCut *Xho* I restriction enzyme (TaKaRa, Beijing, China). After purification, the ORF of *GmHS1^pro-1^* was inserted into the linearized pNI-GmUbi plasmid with an In-Fusion Snap Assembly Mix (TaKaRa, Beijing, China).

### 2.7. RNA Extraction and Real-Time qPCR

A real-time qPCR assay was used to test the expression levels of *GmHS1^pro-1^* in the FHRs and the empty vector FHR control. The total RNA was isolated from different FHR tissues with an RNAiso Plus reagent (TaKaRa, Beijing, China) and cDNA was synthesized using a PrimeScript™ RT reagent kit (TaKaRa, Beijing, China). Real-time qPCR was performed on a CFX Connect qPCR system (Bio-rad, San Francisco, CA, USA) according to the manufacturer’s instructions with a ChamQ SYBR qPCR mix (Vazyme, Nanjing, China). The primers for the RT-qPCR are listed in Supplementary Materials Table S2. The calculation of the relative expression level followed the Livak method [30].

### 2.8. Detection of Phytohormones

The soybean root tissues with overexpressed *GmHS1^pro-1^* (OE) and the wild-type soybean Williams 82 (CK) were obtained and frozen with liquid nitrogen. The indoleacetic acid (IAA), jasmonic acid (JA), salicylic acid (SA) and gibberellin (GA) contents were then detected by MetWare (http://www.metware.cn/, accessed on 29 October 2020) based on the AB Sciex QTRAP^®^ 6500+ LC-MS/MS System. Three replicates of each assay were performed.

## 3. Results

### 3.1. Construction of the Expression Vector for Transformation

Previous studies showed that the activity of the CaMV 35S promoter was low in soybean hairy roots [31]. To induce hairy roots with a robust fluorescence, we constructed a binary vector with a GFP cassette and it was named pNI-GmUbi. The T-DNA structure of pNI-GmUbi is shown in Figure 1a. In the GFP cassette, the *egfp* gene was driven by the CsVMV promoter; in another cassette, he GmUbi promoter was inserted to promote the target gene expression. Both promoters confirmed a higher promoter activity in soybeans [24].

### 3.2. Establishment of the FHR-SCN Pathosystem

The processes of cultivation and the induction of the soybean hairy root were as follows: the soybean seeds were placed into wet vermiculite for germination (Figure 1b); 5-day-old seedlings were harvested to prepare the explants (Figure 1c); a bacterial mass of *A. rhizogenes* strain K599 was collected from an overnight cultured LB plate with a spreader for inoculation (Figure 1d); the seed coats and hypocotyls of the healthy seedlings were removed (Figure 1e) and the eligible explant is shown in Figure 1f; a drop of the bacterial mass was inoculated onto the wound site of the explants (Figure 1g); the explants were vertically inserted into wet vermiculite (Figure 1h); and the cup was covered with Petri dishes or a plastic cup and sealed with plastic wrap to maintain a high humidity (Figure 1i). Finally, 20 days after the *A. rhizogenes* inoculation, the positive hairy roots were identified via a blue light flashlight and a bind pass filter (λ_0_ ≈ 495 nm).

**Figure 2:**
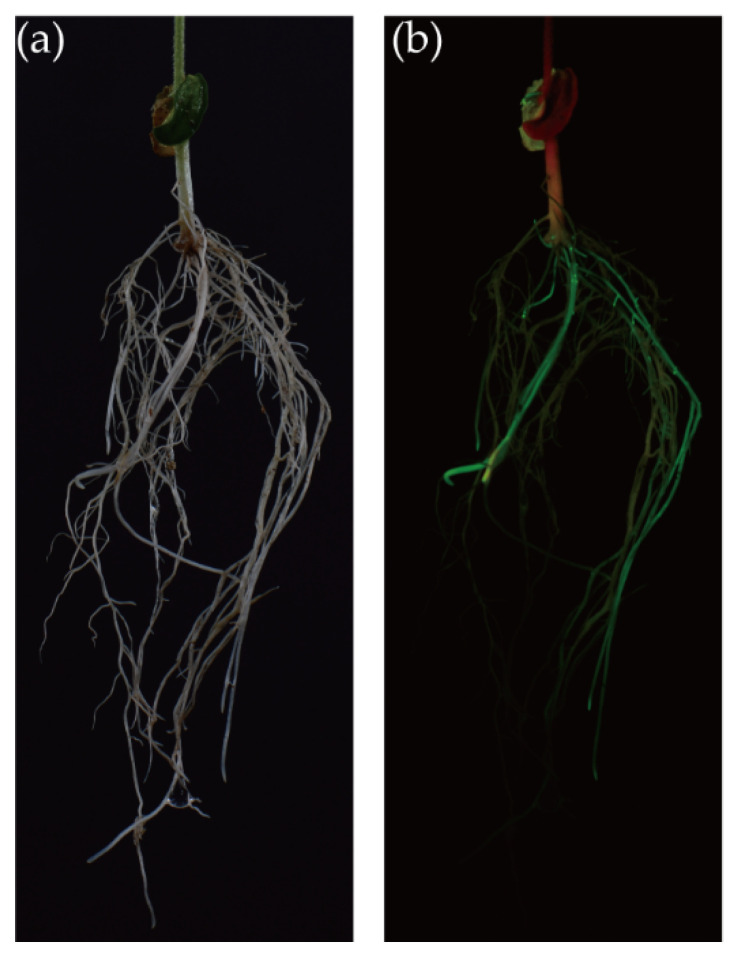
Root system regenerated from the wound of explant. (**a**) Root system observed by a bright-field where the adventitious roots and positive hairy roots cannot be separated by sight; (**b**) root system observed by a 495 nm bind pass filter where the adventitious roots and fluorescent hairy roots can be easily distinguished with LUYOR-3415RG used as the excitation light source.

Figure 2a,b show the regenerated soybean roots and corresponding fluorescent positive hairy roots. To measure the transformation efficiency and frequency, we counted the numbers of roots and GFP-tagged roots in the composite plants. As a result, 93.3% of the W82 seedlings produced FHRs 20 days after the inoculation with *A. rhizogenes* strain K599 (*n* = 30). Additionally, the number of total hairy roots and FHRs of each positive seedling were 10.71 ± 2.27 and 3.79 ± 1.50, respectively (*n* = 28); the positive rate was 34.1%. Among the 28 composite plants, 23 regenerated at least 3 FHRs, which were collected for further experiments.

**Figure 3:**
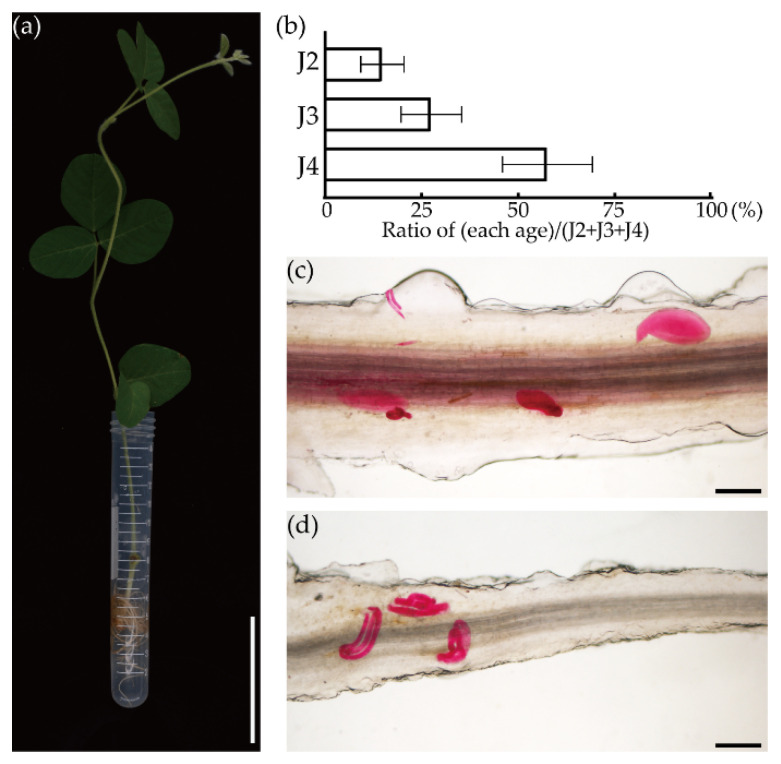
SCN inoculation in FHRs and the demographics assay. (**a**) FHRs were soaked in water-agar with 500 J2s. Bar = 5 cm; (**b**) population of SCNs at different developmental stages in FHRs at 12 dpi; (**c**) females and males (**d**) in FHRs. Bars = 200 μm.

SCN race 3 was used as the pathogen and the processes were as follows: to inoculate SCNs into the FHRs, 500 living J2 SCNs were gently resuspended in 5 mL of a 0.2% water-agar medium and returned to the bottom of a 15 mL centrifuge tube. The adventitious laterals roots and non-fluorescent hairy roots were removed and all of the FHRs were soaked in water-agar (Figure 3a). The plants were grown under a weak light and the roots were kept away from light for 2 days (2 days post-inoculation, 2 dpi) and stained at 12 dpi to calculate the nematode development. As a result, the numbers of nematodes in the different stages were 5.5 ± 2.12 (J2), 10.38 ± 4.12 (J3) and 20.75 ± 3.86 (J4 and adults) on average (*n* = 8) and 85.14% ± 5.23% of SCNs developed at 12 dpi in FHRs (Figure 3b). Moreover, male and female SCNs could be found in the roots (Figure 3c,d). These results indicated the compatibility between SCN race 3 and FHRs.

Compared with the genetic transformation, FHR-SCN demonstrated its efficiency and rapidity in verifying the function of the resistance candidate genes within 6 weeks. In general, the results showed that FHR-SCN is an important method that provided a visual screening methodology in studying the interaction between soybeans and SCNs.

### 3.3. Extension of the Application of the FHR-SCN Pathosystem in Multiple Cultivars

**Figure 4:**
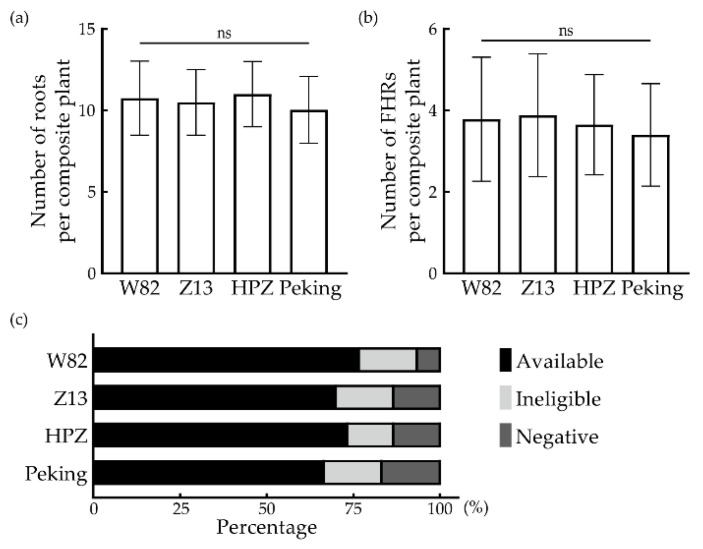
Total number of roots (**a**) and FHRs (**b**) per composite plant in different soybean cultivars. (**a**) Number of total roots (one-way ANOVA); (**b**) number of FHRs (one-way ANOVA). Values are mean ± SD of at least 20 biological replications, *p* > 0.05 means no significant (ns); (**c**) available rate for SCN inoculation in different soybean cultivars where the definition is that the number of FHRs is not less than three.

Zhonghuang 13 (Z13), Huipizhiheidou (HPZ, ZDD 2315) and Peking are also widely used in the study of the soybean–SCN interaction [3,32]. To expand the application of the FHR-SCN system, we further analyzed the transformation efficiency in these cultivars; 30 explants were selected from each cultivar for FHR induction and the numbers of the total hairy roots were 10.50 ± 1.99 (*n* = 26), 11.00 ± 1.96 (*n* = 26) and 10.04 ± 2.01 (*n* = 25), respectively (Figure 4a). The number of regenerated FHRs were 3.88 ± 1.48 (*n* = 26), 3.65 ± 1.21 (*n* = 26) and 3.40 ± 1.23 (*n* = 25), respectively (Figure 4b). No significant difference was observed among these cultivars.

As mentioned above, we selected composite plants that regenerated at least 3 FHRs for SCN inoculation. It was necessary to calculate the available ratio to estimate the number of explants in the experiments. The result showed that 23, 22, 21 and 20 available composite plants were collected in W82, Z13, HPZ and Peking; the available ratios of them were 76.67%, 70%, 73.33% and 66.67%, respectively (Figure 4c).

### 3.4. Overexpression of GmHS1^pro-1^ Enhances Resistance against H. Glycines Race 3

There are two main methods for plants to resist parasitic nematodes; one is inhibiting the nematode infection and the other is inhibiting the nematode development. Studies have shown that an overexpression *HS1^pro-1^* suppressed the development of the beet cyst nematode (BCN, *Heterodera schachtii* Schm.) [33,34,35]. *HS1^pro-1^* was firstly cloned from *Beta procumbens* and its homologous gene in soybean, *GmHS1^pro-1^*, was also considered to have a resistance potential against the soybean cyst nematode. However, functional studies are lacking.

Thus, we evaluated the resistant phenotype of *GmHS1^pro-1^* in Williams 82 hairy roots. *GmHS1^pro-1^* was transferred into the expression vector, pNI-GmUbi, and a transgenic FHR (FHR-OE) was induced. Williams 82 FHRs with empty pNI-GmUbi were also induced as a control (FHR-EV).

**Figure 5:**
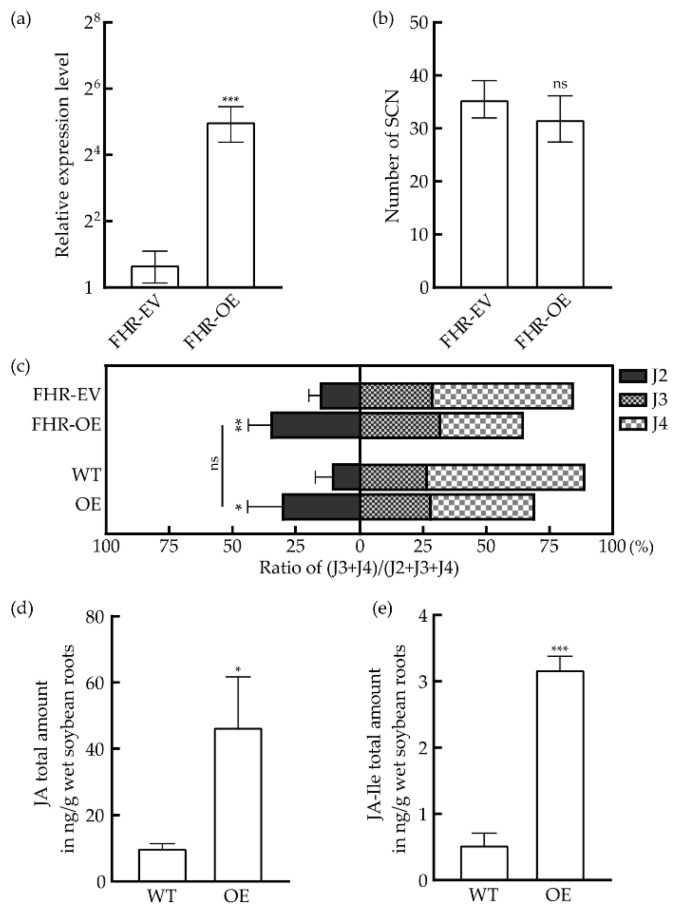
*GmHS1^pro-1^* suppressed the development of *H. glycines* by increasing the JA content. (**a**) Relative expression levels of *GmHS1^pro-1^* in overexpressed FHRs (FHR-OE, *n* = 8) and empty vector control (FHR-EV, *n* = 7) (Student’s *t*-test, *p* < 0.001); (**b**) total number of SCNs in FHRs that overexpressed *GmHS1^pro-1^* (FHR-OE, *n* = 9) and empty vector control (FHR-EV, *n* = 6) (Student’s *t*-test, *p* = 0.105); (**c**) demographics assays of SCNs in FHRs that overexpressed *GmHS1^pro-1^* (FHR-OE, *n =* 9), empty vector control (FHR-EV, *n* = 6), *GmHS1^pro-1^* transgenic soybean lines (OE, *n* = 7) and Williams 82 wild-type control (WT, *n* = 6) (one-way ANOVA, *p* = 0.006 in FHR-EV versus FHR-OE, *p* = 0.013 in WT versus OE); (**d**) JA content in WT and *GmHS1^pro-1^* overexpressed soybeans (Student’s *t*-test, *p* = 0.016, *n* = 3); (**e**) JA-Ile content in WT and *GmHS1^pro-1^* overexpressed soybeans (Student’s *t*-test, *p* < 0.001, *n* = 3). Data are means ± SD. Data are means ± SD, *p* > 0.05 means no significant (ns), *p* < 0.05 is considered statistically significant (*** *p* < 0.001; ** *p* < 0.01; * *p* < 0.05).

In the positive FHRs (FHR-OE, *n* = 8), the relative expression level of *GmHS1^pro-1^* was 20.04 ± 6.65 times higher than its empty vector control (FHR-EV, *n* = 7); the result is shown in Figure 5a. However, there was no significant difference between FHR-OE (*n* = 9) and FHR-EV (*n* = 6) in the number of nematodes, which suggested that the overexpression of *GmHS1^pro-1^* may not inhibit SCN invasion into soybean roots (Figure 5b). Interestingly, the developmental stage of SCNs in FHR-OE was slower, which was reflected in the increase percentage of J2 and the decrease of J3 and J4 (Figure 5c).

We reproduced this result with transgenic Williams 82 soybean plants, which expressed *GmHS1^pro-1^* (OE, *n* = 7) and the wild-type Williams 82 control (WT, *n* = 5, Figure 5c). There was no significant difference in the percentage of J2 in the roots between FHR-OE and the transgenic plants (OE). The result indicated that the same phenotype could be observed with FHRs and the genetic transformation.

Previous studies have shown that SA and JA played positive roles in resistance regulation; the SCN resistance increased after the treatment of JA or SA in soybeans [6]. Based on these results, we speculated that the overexpression of *GmHS1^pro-1^* might affect the phytohormone levels in soybeans. To validate this, we then assayed the content of IAA, JA, SA and GA. Compared with the WT group, we found the content of JA and JA-Ile increased in plants that overexpressed *GmHS1^pro-1^* (Figure 5d,e). The detected compounds and contents in this experiment are provided in Supplementary Materials Table S3. These results indicated that *GmHS1^pro-1^* might regulate resistance through the JA pathway.

## 4. Discussion

### 4.1. The FHR-SCN Pathosystem Is a Rapid, Efficient and Visible Method for the Verification of Resistance Candidate Genes against SCNs

In recent years, the hairy root has been widely used in soybeans and other crops. Jiang et al. [36] developed a soybean hairy root versus a soybean mosaic virus pathosystem (ALRSHR-SMV) to study the interaction between soybeans and SMVs. Previous studies identified SCN resistance genes using hairy root systems to reduce soybean yield losses [7]. The detection of transgenic roots depends on PCR or Sanger sequencing and is a cumbersome verification process. In the present study, the FHR-SCN pathosystem was established and could be used to visualize the screening of positive hairy roots. The FHR-SCN pathosystem was optimized to evade most of the aseptic operation, which reduced the probability of failure. Furthermore, the FHR-SCN pathosystem only needed approximately six weeks to verify the resistance phenotype of the candidate gene against SCNs. Additionally, it could be used with many cultivars and there was no significant difference in the number of induced FHRs among them (Figure 3). The demographic assays of SCNs are always cumbersome. We hope that the FHR-SCN pathosystem can accelerate the research of interactions between soybeans and nematodes.

### 4.2. The Application of FHRs in Other Assays

Based on the results, it could be speculated that the FHR-SCN could be used in more reverse genetic assays including RNAi and CRISPR/Cas [6]. Previous studies have demonstrated the application of soybean hairy roots in subcellular localization, bi-molecular fluorescence complementation (BiFC) and fluorescence resonance energy transfer (FERT) [6]. These studies have enhanced our confidence in the application of FHR to more assays such as co-immunoprecipitation (CoIP) and luciferase assays. To facilitate the subsequent modification of the plasmid, we added digestion sites on pNI-GmUbi (Supplementary Materials Figure S1).

### 4.3. Key Steps That Affect the Transformation Efficiency

Several steps and manipulations may affect the efficiency of the FHR regeneration. The first step is to use a fresh bacterial carpet because overcultured bacteria will reduce the efficiency [18]. Secondly, as described in a previous study [37], bacterial suspensions can be added into vermiculite to enhance the infection efficiency but too much added medium may cause the yellowing of the soybean explant. A high humidity is critical for FHR regeneration. We do not recommend adding too much nutrient solution into the vermiculite because excessive irrigation may lead to abnormal root growth. The last step, the SCNs—especially J2s—are very vulnerable; the inoculation of J2s should be gentle.

### 4.4. GmHS1^pro-1^ Suppresses the Development of H. Glycines

Our research showed that the upregulation of *GmHS1^pro-1^* increased the contents of JA and JA-Ile. The overexpression of *Arabidopsis* JA biosynthesis genes, *AtNPR1*, *AtTGA2* and *AtPR-5*, provides a modest resistance to SCNs in soybeans [38]. In contrast, JA biosynthesis genes were downregulated in susceptible soybeans under an SCN infection [39]. In *Arabidopsis*, the increase of JA and JA-Ile is beneficial for resistance against root-knot nematodes (*Meloidogyne hapla*) [40]. Together, these results indicate that the JA pathway and content are positively correlated with resistance against parasitic plant nematodes. Based on this, we strongly suggest that the overexpression of *GmHS1^pro-1^* suppressed the development of SCNs through the active JA pathway.

Further, the overexpression of *GmHS1^pro-1^* showed no contribution to reducing the number of SCNs invading soybean roots because plants mainly resist nematode infections by improving the mechanical strength of the roots.

## 5. Conclusions

In this study, we established a pathosystem, FHR-SCN, to verify the functional genes in soybeans and the SCN interaction. With this method, fluorescent hairy roots could be induced in 25 days. At least two-thirds of them could be used for SCN inoculation. A demographics analysis of the SCN in FHRs could be counted at 12 dpi. In summary, the phenotype of the candidate gene could be verified within 6 weeks via this method. In order to verify this method, *GmHS1^pro-1^* was overexpressed and the results illustrated that *GmHS1^pro-1^* provided additional resistance in both FHRs and transgenic soybeans. We further explained the mechanism of *GmHS1^pro-1^* and the results suggested that the increase of JA and JA-Ile may contribute to the resistance.

## Data Availability

Not applicable.

## Supplementary Materials

The following are available online at https://www.mdpi.com/article/10.3390/biology10121353/s1, Figure S1: A diagram of the pNI-GmUbi plasmid and the illustration of construction. Table S1: Primer pairs for cloning *GmHS1^pro-1^*. Table S2: Primer pairs for qPCR assay. Table S3: Phytohormone content in OE and WT plants.
